# CHAPLE‐Like Enteropathy and Renal Microangiopathy in an Adult Patient With CD46 Deficiency

**DOI:** 10.1002/jcla.70370

**Published:** 2026-10-06

**Authors:** Bircan Erden, Pelin Korkmaz, Halil Yazici, Ahmet Burak Dirim, Fatih Beşışık, Nevzat Kahveci, Zeynep Kilinc, Derya Unal, Osman Ozan Yegit, Tuğba Kalaycı, Özge Hürdoğan, Yasemin Özlük, Melek Büyük, Asli Gelincik, Semra Demir

**Affiliations:** ^1^ Division of Allergy and Immunology, Department of Internal Medicine, Istanbul Faculty of Medicine Istanbul University Istanbul Türkiye; ^2^ Division of Nephrology, Department of Internal Medicine, Istanbul Faculty of Medicine Istanbul University Istanbul Türkiye; ^3^ Division of Gastroenterology, Department of Internal Medicine, Istanbul Faculty of Medicine Istanbul University Istanbul Türkiye; ^4^ Division of Medical Genetics, Department of Internal Medicine, Istanbul Faculty of Medicine Istanbul University Istanbul Türkiye; ^5^ Department of Pathology, Istanbul Faculty of Medicine Istanbul University Istanbul Türkiye

**Keywords:** CD46, CHAPLE, complement, eculizumab, hypogammaglobulinemia, intestinal lymphangiectasia, membrane cofactor protein, membranous nephropathy, protein‐losing enteropathy, thrombotic microangiopathy

## Abstract

**Background:**

Protein‐losing enteropathy is a rarely reported feature of CD46‐associated disease and may resemble CD55 deficiency with hyperactivation of complement, angiopathic thrombosis, and protein‐losing enteropathy (CHAPLE) syndrome. Associated hypogammaglobulinemia may be mistaken for primary antibody deficiency.

**Methods:**

We describe a 23‐year‐old woman with childhood‐onset enteropathy and hypogammaglobulinemia who had received immunoglobulin replacement for presumed common variable immunodeficiency since age 5. Multidisciplinary reassessment incorporated intestinal and renal histopathology, whole‐exome sequencing, family segregation, and longitudinal evaluation of the response to eculizumab.

**Results:**

Duodenal biopsy demonstrated intestinal lymphangiectasia, whereas kidney biopsy revealed chronic thrombotic microangiopathy with concurrent PLA2R‐negative membranous nephropathy. Serum C3 and C4 were within reference ranges. Sequencing identified homozygous CD46 c.565T>G (p.Tyr189Asp), with no pathogenic CD55 variant. The diagnostic laboratory reported the CD46 finding as a variant of uncertain significance. Family segregation and published functional evidence supported its clinical relevance, although patient‐specific functional confirmation was unavailable. Eculizumab promptly improved abdominal pain, vomiting, and oral intake. By April 2026, following dose escalation, serum albumin reached 4.3 g/dL and IgG 554 mg/dL, whereas proteinuria decreased from 5.95 to 2.4 g/day.

**Conclusion:**

This case supports considering CD46‐associated disease in adults with a CHAPLE‐like phenotype, particularly when enteric protein loss and hypogammaglobulinemia coexist with renal microangiopathy despite normal C3 and C4. The response to complement blockade supports a complement‐mediated disease process. Documented protein loss should prompt reassessment of a presumptive diagnosis of primary antibody deficiency.

## Graphical abstract

We report an adult patient with a homozygous CD46 variant presenting with lifelong protein‐losing enteropathy, intestinal lymphangiectasia, and CVID‐like hypogammaglobulinemia mimicking CHAPLE syndrome. Concurrent renal biopsy revealed PLA2R‐negative membranous nephropathy with chronic thrombotic microangiopathy, establishing multi‐organ complement‐mediated injury. Terminal complement blockade with eculizumab led to prompt improvement in gastrointestinal protein loss, underscoring CD46 as a diagnostic alternative in CHAPLE‐like presentations with negative CD55 sequencing.

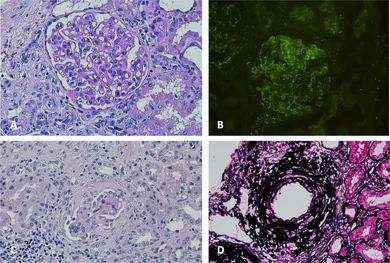

## 1 Case Report

CD46 (membrane cofactor protein) and CD55 (decay‐accelerating factor) are nonredundant membrane complement regulators [1]. Biallelic CD55 loss causes CHAPLE syndrome, characterized by childhood‐onset intestinal lymphangiectasia, hypoalbuminemia, and thrombosis [2]. CD46 variants are classically associated with complement‐mediated thrombotic microangiopathy (TMA) and atypical hemolytic uremic syndrome (aHUS) [3, 4]; protein‐losing enteropathy (PLE) was only recently reported [5]. To our knowledge, this is the first reported CD46‐associated CHAPLE‐like PLE diagnosed and treated in adulthood.

A 23‐year‐old woman, born to consanguineous parents, had near‐daily abdominal pain relieved by vomiting from birth and episodic non‐bloody diarrhea after complementary feeding. At age 5, evaluation for growth failure and diarrhea showed elevated fecal alpha‐1 antitrypsin, hepatosplenomegaly, and hypogammaglobulinemia (IgG 156 mg/dL [700–1600], IgA 41 mg/dL [70–400], and IgM 48 mg/dL [40–230]). She received a presumptive diagnosis of common variable immunodeficiency (CVID) and started immunoglobulin replacement therapy (IgRT). Pre‐IgRT tetanus‐ and pneumococcal‐specific responses were undocumented [6]. A targeted CVID‐PAD 27‐gene panel was negative.

In early adolescence, she developed hypertension, microscopic hematuria, and low‐grade proteinuria. She transferred to adult immunology care in January 2022 at age 18, without recurrent sinopulmonary or opportunistic infections but with persistent gastrointestinal symptoms, poor weight gain, cachexia, pallor, and mild splenomegaly. IgRT continued while multidisciplinary reassessment began.

In 2023, at age 20, she returned with progressive edema, palpitations, vomiting, severe microcytic anemia, and pleural effusion. Computed tomography showed diffuse small‐bowel wall thickening; albumin was 2.41 g/dL and IgG 174 mg/dL despite IgRT. Endoscopy and duodenal biopsy showed intestinal lymphangiectasia with chronic inflammation. Renal evaluation showed progressive proteinuria with persistent microscopic hematuria. Kidney biopsy demonstrated PLA2R‐negative membranous nephropathy along with hyperplastic changes within arterioles and ischemic glomerular changes, consistent with chronic thrombotic microangiopathy (TMA) (Figure 1). Multidisciplinary nephropathology review considered chronic TMA the dominant renal lesion; extended membranous‐nephropathy antigen testing was not performed. Available serum C3 and C4 measurements were within reference ranges; CH50, AH50, and factor H, I, and B were unavailable.

**FIGURE 1:**
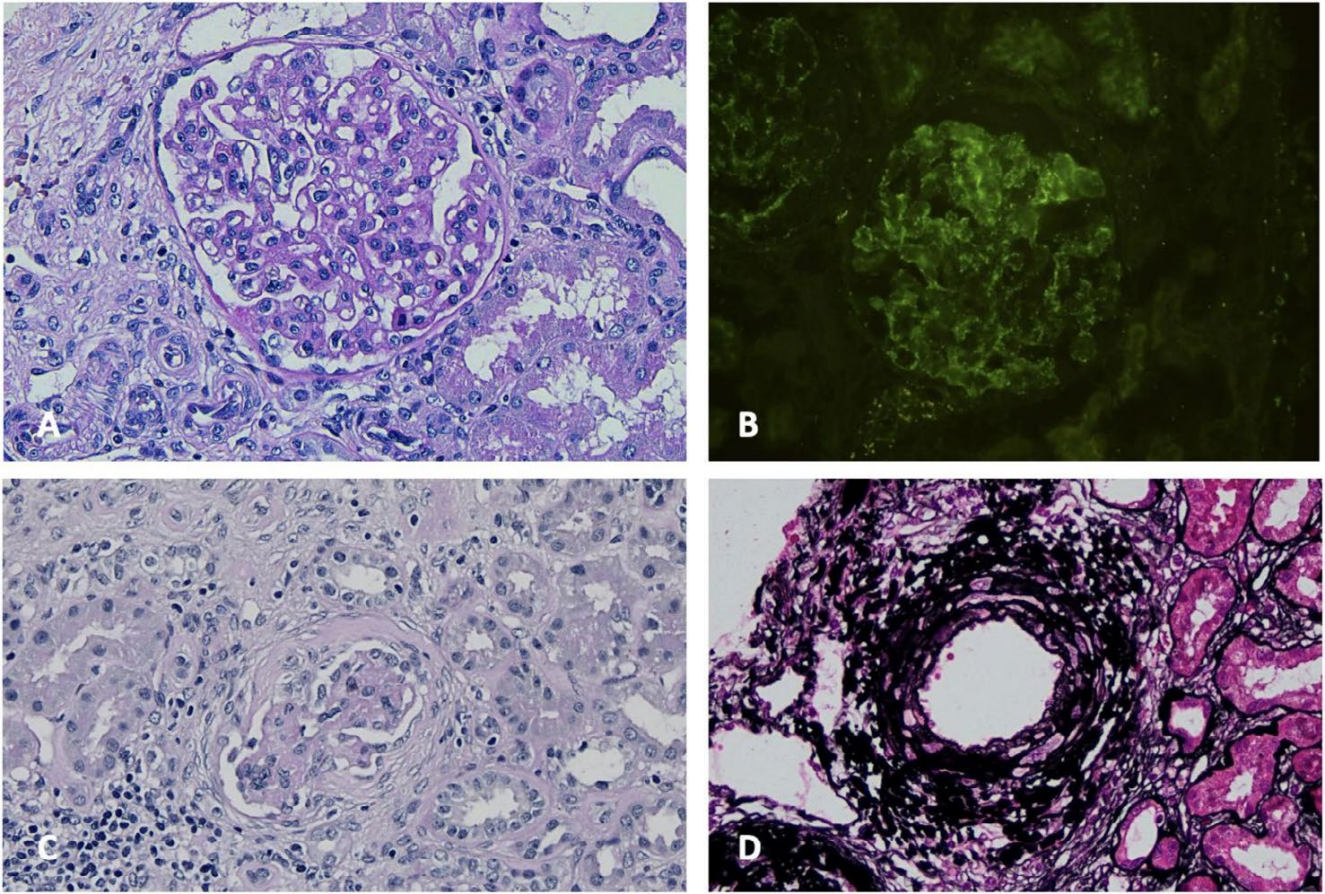
Histopathological findings of renal involvement. (A) A glomerulus showing diffuse thickening and segmental duplication of the basement membranes (Periodic acid–Schiff stain, ×400). (B) Diffuse granular, strong IgG deposition along the basement membranes on immunofluorescence examination (anti‐IgG antibody, direct immunofluorescence, ×400). (C) An ischemic glomerulus demonstrating narrowing of capillary lumina (Hematoxylin and eosin, ×400). (D) Hyperplastic changes in an arteriole section (Jones methenamine silver, ×400).

Whole‐exome sequencing (requested December 2023; reported August 2024) found no pathogenic CD55 variant and identified homozygous CD46 c.565 T>G (p.Tyr189Asp; rs202071781), classified as a variant of uncertain significance by the diagnostic laboratory. It is rare in population databases (aggregate frequency approximately 1.6e‐5; gnomAD up to 0.005%) and has a published CADD Phred score of 23.8; ClinVar classifications are conflicting, and four of five summarized in silico tools predict a damaging effect [7, 8]. Published studies showed non‐detectable C3b/C4b binding and negligible factor I cofactor activity [4, 5]. No additional pathogenic or likely pathogenic coding variant was found in evaluated TMA genes, including CFH, CFI, C3, CFB, THBD, and CFHR1‐5; dedicated CFH/CFHR structural analysis was not performed. Both parents were heterozygous; an older sister with HUS was homozygous. She did not attend further assessment and was reportedly asymptomatic outside HUS episodes; subclinical PLE or hypogammaglobulinemia could not be excluded. Patient‐specific CD46 flow cytometry was not performed.

After meningococcal vaccination, eculizumab was initiated on 12 February 2025 at age 21. Following consultation with a CHAPLE‐experienced center, an individualized lower‐dose regimen was used because of the CHAPLE‐like phenotype and preserved creatinine (600 mg weekly for 4 weeks, then 900 mg every 2 weeks), with planned escalation for incomplete response. Abdominal pain, vomiting, and oral intake improved after the first dose. By April 2025, albumin had increased to 3.17 g/dL, IgG to 310 mg/dL, and hemoglobin to 10.4 g/dL. On 12 May 2025, nephrotic‐range proteinuria persisted (5.95 g/day; creatinine 0.46 mg/dL), and biochemical response remained incomplete. By 26 May 2025, she had received nine doses. Treatment was subsequently escalated to the standard adult aHUS maintenance dose of 1200 mg every 2 weeks [9]. By April 2026, after dose escalation, 24‐h urinary protein excretion had decreased to 2.4 g/day, while serum albumin increased to 4.3 g/dL and IgG to 554 mg/dL.

Because CD46 regulates surface‐bound C3b/C4b, normal circulating C3 and C4 do not exclude dysfunction [1, 4]. Absent functional complement profiling and patient‐specific CD46 expression are limitations. TMA and response to C5 blockade support complement‐mediated disease but are not CD46‐specific; segregation and published functional evidence strengthen attribution.

The initial CVID label was misleading because enteric protein loss was already evident and specific antibody responses were not documented before IgRT [6]. Although CD46 has immunomodulatory functions [10], improvement in albumin and IgG after complement blockade supports a major secondary enteric‐loss component.

Eculizumab is established for complement‐mediated TMA/aHUS [3, 11]. In CD55‐associated CHAPLE, C5 blockade improves PLE and systemic manifestations [12]. The pediatric patient with the same variant had nearly absent PBMC CD46 expression and PLE resolution after eculizumab [5]. In our patient, gastrointestinal improvement preceded complete biochemical or renal response; the PLA2R‐negative membranous pattern remained concurrent.

CD55‐negative CHAPLE‐like presentations should prompt broader complement‐regulatory evaluation, particularly when hypogammaglobulinemia coexists with renal microangiopathy. This case expands CD46‐related disease and supports reassessing apparent CVID when objective protein loss is present.

## Funding

The authors have nothing to report.

## Ethics Statement

Ethics committee approval was not required for this single case report according to local regulations.

## Consent

Written informed consent for publication of this case report, including any accompanying clinical images, was obtained from the patient.

## Conflicts of Interest

The authors declare no conflicts of interest.

## Data Availability

Data supporting the findings of this report are available from the corresponding author upon reasonable request.

## References

[1] M. K. Liszewski and J. P. Atkinson , “Membrane Cofactor Protein (MCP; CD46): Deficiency States and Pathogen Connections,” Current Opinion in Immunology 72 (2021): 126–134, 10.1016/j.coi.2021.04.005. PMC8123722 34004375

[2] A. Ozen , W. A. Comrie , R. C. Ardy , et al., “CD55 Deficiency, Early‐Onset Protein‐Losing Enteropathy, and Thrombosis,” New England Journal of Medicine 377, no. 1 (2017): 52–61, 10.1056/NEJMoa1615887. PMC6690356 28657829

[3] M. Noris and G. Remuzzi , “Atypical Hemolytic‐Uremic Syndrome,” New England Journal of Medicine 361, no. 17 (2009): 1676–1687, 10.1056/NEJMra0902814. 19846853

[4] V. Fremeaux‐Bacchi , E. A. Moulton , D. Kavanagh , et al., “Genetic and Functional Analyses of Membrane Cofactor Protein (CD46) Mutations in Atypical Hemolytic Uremic Syndrome,” Journal of the American Society of Nephrology 17, no. 7 (2006): 2017–2025, 10.1681/ASN.2005101051. 16762990

[5] C. Wang , J. Chen , X. Han , et al., “Protein‐Losing Enteropathy as a New Phenotype in Atypical Hemolytic Uremic Syndrome Caused by CD46 Gene Mutation,” Pediatric Nephrology 39, no. 12 (2024): 3513–3520, 10.1007/s00467-024-06451-0. 39097532

[6] F. A. Bonilla , I. Barlan , H. Chapel , et al., “International Consensus Document (ICON): Common Variable Immunodeficiency Disorders,” Journal of Allergy and Clinical Immunology. In Practice 4, no. 1 (2016): 38–59, 10.1016/j.jaip.2015.07.025. PMC4869529 26563668

[7] R. Piras , P. Iatropoulos , E. Bresin , et al., “Molecular Studies and an Ex Vivo Complement Assay on Endothelium Highlight the Genetic Complexity of Atypical Hemolytic Uremic Syndrome: The Case of a Pedigree With a Null CD46 Variant,” Frontiers in Medicine 7 (2020): 579418, 10.3389/fmed.2020.579418. PMC7670076 33224962

[8] National Center for Biotechnology Information , “ClinVar; NM_172351.3(CD46):c.565T>G (p.Tyr189Asp), Variation ID 988106, VCV000988106.12,” accessed August 13, 2026.

[9] Alexion Pharmaceuticals, Inc , “SOLIRIS® (Eculizumab) Injection, for Intravenous Use,” Prescribing Information, DailyMed, U.S. National Library of Medicine, Revised [ay/yıl], accessed [erişim tarihi, ör. September 27, 2026], https://dailymed.nlm.nih.gov/dailymed/lookup.cfm?setid=ebcd67fa‐b4d1‐4a22‐b33d‐ee8bf6b9c722.

[10] A. Fuchs , J. P. Atkinson , V. Fremeaux‐Bacchi , and C. Kemper , “CD46‐Induced Human Treg Enhance B‐Cell Responses,” European Journal of Immunology 39, no. 11 (2009): 3097–3109, 10.1002/eji.200939392. PMC2856319 19784949

[11] C. M. Legendre , C. Licht , P. Muus , et al., “Terminal Complement Inhibitor Eculizumab in Atypical Hemolytic‐Uremic Syndrome,” New England Journal of Medicine 368, no. 23 (2013): 2169–2181, 10.1056/NEJMoa1208981. 23738544

[12] A. Kurolap , O. Eshach Adiv , T. Hershkovitz , et al., “Eculizumab Is Safe and Effective as a Long‐Term Treatment for Protein‐Losing Enteropathy due to CD55 Deficiency,” Journal of Pediatric Gastroenterology and Nutrition 68, no. 3 (2019): 325–333, 10.1097/MPG.0000000000002198. 30418410

